# Antibacterial Effects of Leaf Extract of* Nandina domestica* and the Underlined Mechanism

**DOI:** 10.1155/2018/8298151

**Published:** 2018-01-18

**Authors:** Zhao-Yu Guo, Zhuo-Yang Zhang, Jia-Qi Xiao, Jin-Hong Qin, Wei Zhao

**Affiliations:** ^1^Lab of Medical Microbiology and Parasitology, Experimental Teaching Center of Basic Medicine, Shanghai Jiao Tong University School of Medicine, Shanghai 200025, China; ^2^Department of Medical Laboratory, Shanghai Mental Health Center, Shanghai Jiao Tong University School of Medicine, Shanghai 200030, China; ^3^Department of Immunology and Medical Microbiology, Shanghai Jiao Tong University School of Medicine, Shanghai 200025, China

## Abstract

**Aim:**

The study was conducted to investigate the antibacterial and antiasthmatic effects of* Nandina domestica* leaf extract, to find out its active components, and to assess its safety issue.

**Methods:**

(1) Solid-phase agar dilution method was used for antibacterial activity test of nandina leaf extract and the change of bacterial morphology after treatment was observed under the transmission microscope; (2) guinea pig model of asthma was used to test the asthma prevention effect of nandina leaf extract; (3) alkaloids and flavones were separated from nandina leaf extract and were further analyzed with HPLC-MS; (4) mice model was used to assessment of the safety issue of nandina leaf extract.

**Results:**

(1) Nandina leaf extract inhibited the growth of bacteria and destroyed bacterial membrane; (2) nandina leaf extract alleviated animal allergy and asthma; (3) the components reextracted by ethyl acetate were active, in which alkaloids inhibited Gram-positive bacteria and prevented asthma and flavones inhibited Gram-negative bacteria; (4) nandina leaf extract had no toxic effect on mice.

**Conclusion:**

Nandina leaves inhibit bacterial growth and prevent asthma through alkaloids and flavones, which had integrated function against chronic bronchitis. This study provided theoretical basement for producing new Chinese medicine against chronic bronchitis.

## 1. Introduction

*Nandina domestica* is a suckering shrub in the Berberidaceae family, which grows along Yangtze River. The decoction of medicinal herbs from nandina leaves has been used to treat chronic tracheitis in Chinese medicine. In this study, we tried to find out if nandina leaves have activities to prevent asthma and to inhibit bacteria growth. The results showed that all samples can reduce the symptom of the asthma and can inhibit the growth of bacteria. Further study showed that nandina had cell toxic effect. Since nandina functions very differently from other antibiotics, our result will shed light on how to exploit nandina for treating superbacteria. Alkaloids and flavones were found in nandina leaf extract and detailed components were identified by HPLC-MS/MS. The effect of nandina leaf extract on asthma might be due to these alkaloids and flavones.

## 2. Background

*Nandina domestica*, in the family of Berberidaceae, originates from China and Japan. It is a suckering shrub, about 2 m high, with straight stem and a few branches. Its new branches are pink, and its leaves grow in pairs. It blooms in summer with white or pink flowers [1–3]. The decoction of medicine herbs from nandina leaves has been used to treat chronic bronchitis in Chinese medicine. The mother of one of my classmates has suffered for a long time from chronic bronchitis and her symptom alleviates after treatment by nandina leaf extract.

Chronic bronchitis is usually caused by infection (*Staphylococcus*,* Pseudomonas*,* Acinetobacter*,* Streptococcus*, and* Escherichia*) [4, 5], followed by chronic nonspecific inflammation of mucosa in trachea, bronchus, and the surrounding tissue. Clinical symptoms include repetitive coughing, expectoration, and asthma. The pathogenesis of chronic bronchitis is complicated and is still unknown yet. The commonly used methods to treat chronic bronchitis include controlling infection, preventing asthma and expectorant, and reducing coughing.

We supposed that the extract of nandina leaves may have an effect on bacteria inhibition and on asthma prevention. The active components of nandina leaf extract were further investigated as well as its safety issue assessment. This study provided the scientific foundation for the exploiting nandina against chronic bronchitis.

## 3. Materials and Methods

### 3.1. Materials

#### 3.1.1. Nandina Leaves

They were collected from Wujing Town in Shanghai and were identified as* Nandina domestica* Thunb. by Professor Luping Qin from Shanghai Secondary Military Medical university.

#### 3.1.2. Bacteria Strains

*Staphylococcus aureus* ATCC25923,* Escherichia coli* ATCC25922,* Streptococcus pyogenes* CMCC(B)32175,* Pseudomonas aeruginosa* ATCC25793, and* Acinetobacter baumannii* JMD80 were provided by Department of Molecular Microbiology, Shanghai Jiao Tong University School of Medicine.

#### 3.1.3. Animals

Kunming mice and guinea pigs were raised in clean level cabinet in Department of Experimental Zoology, Shanghai Jiao Tong University School of Medicine. Kunming mice were 5–7 weeks old, about 20 g, 9 female and 9 male. Guinea pigs were 8 weeks old, about 250–300 g, 30 female and 30 male. 6 mice with equal sexes were used for each group.

### 3.2. Methods

#### 3.2.1. Extraction of Nandina Leaves

200 g nandina leaves was cut into small pieces and soaked in 2,000 mL water for 1 h at room temperature and then boiled for 20 min with low flame. The supernatant were collected by centrifugation at 10,000 rpm for 20 min, and the pellet was boiled again in 500 mL water. Pooled supernatants were evaporated at 80°C to 200 ml (about 1 g/ml) and the final solution was stored at 4°C for usage.

#### 3.2.2. Antibacterial Activities

Solid-phase agar dilution method was used for antibacterial activities. Nandina leaf extract was added to 10 ml prewarmed MH agar medium (at 55°C) to the final concentration of 24, 12, 6, 3, and 1.5 mg/ml and were plated in 9 cm diameter dishes. After solidification, plates were prewarmed at 37°C for 30 min.* Staphylococcus aureus* ATCC25923,* Escherichia coli* ATCC25922,* Streptococcus pyogenes* CMCC(B)32175,* Pseudomonas aeruginosa* ATCC25793, and* Acinetobacter baumannii* JMD80 strains were cultured for 6–8 h before usage. 10 *μ*l of each bacteria strain diluted with saline to 1~9 × 10^5^ cfu/ml was dropped on different parts of the same plate in a 1 cm diameter area. The growth conditions were calculated after 24–48 h of culture. Pure MH medium was used as the negative control. Each experiment was repeated for three times.

#### 3.2.3. Effect of Nandina Leaf Extract on Bacteria Morphology

*Staphylococcus aureus* ATCC25923,* Escherichia coli* ATCC25922,* Streptococcus pyogenes* CMCC(B)32175,* Pseudomonas aeruginosa* ATCC25793, and* Acinetobacter baumannii* JMD80 strains were cultured for 6–8 h before usage. 0.5 ml nandina leaf extract was added to 4.5 ml 1~9 × 10^5^ cfu/ml strains to the final concentration of 24 mg/ml and the treated bacteria were cultured for 30 min and 60 min, respectively, at 37°C. The bacteria were centrifuged and washed twice with saline at 10,000 rpm for 10 min. Then the bacteria were fixed with 2.5% glutaral, dehydrated with gradient ethanol, buried in wax, and stained with uranyl acetate and lead nitrate and observed under HITACHI H-7650 transmission electron microscope.

#### 3.2.4. Activities on Preventing Asthma

Guinea pigs model of asthma was used to test the activity of nandina leaf extract on preventing asthma. Guinea pigs were sensitized by intraperitoneal injection with 10% OVA at 1 ml/kg and were stimulated with 0.5% OVA by aerosol inhalation for four times, 40 s/time on the fourteenth day, and on the next day guinea pigs were restimulated with 1% OVA for 2 min, followed by the third stimulation for 5 min after 4 h. The symptom of guinea pig model of asthma immediately after the last stimulation was recorded. The symptom of guinea pig model of asthma was divided into four degrees: I represents no response; II represents dyspnea; III represents cough; IV represents shock and death. Guinea pigs with symptoms II and III were considered successful guinea pigs model of asthma.

The nandina leaf extract was perfused to guinea pig model of asthma continuously for 16 days at a dose of 1 g/kg/day after the first stimulation. Aminophylline was perfused to guinea pig model of asthma at a dose of 2 mg/kg/day as positive control. The activities of guinea pigs were recorded every day.

#### 3.2.5. Preparation Different Phase of Leaf Extraction

The nandina leaf extract from 2.1 was reextracted with ethanol, petroleum ether, and ethyl acetate, respectively. The solvents from the combined extracts were evaporated by vacuum rotary evaporator into powders and stored at 4°C for future usage. The powders from ethanol, petroleum ether, and ethyl acetate were named extracts A, B, and C, respectively.

#### 3.2.6. The Antibacterial and Asthma Prevention Effect of Extracts A, B, and C

The antibacterial and asthma prevention effects of extracts A, B, and C from 2.5 were evaluated according to 2.3 and 2.4.

#### 3.2.7. Primary Chemical Analysis of Extract C

Dragendorff's reagent and magnesium hydrochloride powder were used to test if there were alkaloids and flavones in extract C, respectively.

#### 3.2.8. Effective Component Groups in Extract C

*(1) Alkaloids in Extract C*. Alkaloids were extracted as Liu et al. [6–8]. Nandina leaf extract was solved in ten volumes of 0.5 mol/L hydrochloride. After filtration, the supernatant was adjusted to pH > 7 with NaOH. Equal amount of dichloromethane was added to the above solution. Mixed solution was shaked clockwise for 5 min and kept still for 5 h. Then the dichloromethane part was collected and condensed. The water part was reextracted with dichloromethane for another 2 times. The concentration of alkaloids was measured according to the previous study [8]. 

*(2) Flavones in Extract C*. Flavones were extracted with the following steps. 1 kg AB-8 resins was activated by acid, base, and ethanol sequentially. Nandina leaf extract was mixed with equal amount of water, mixed, and loaded to the column. The column was then eluted by water, 20%, 40%, and 60% ethanol. The concentration of flavones was measured by ultraviolet spectrophotometer. The elution with concentration higher than 50% was collected and condensed by evaporation. 

*(3) The Antibacterial and Asthma Prevention Effect of Alkaloids and Flavones*. Alkaloids and flavones were diluted to certain concentrations and the effect of alkaloids and flavones were evaluated as 2.3 and 2.4. 

*(4) HPLC-MS Analysis of Effective Compounds in Alkaloids and Flavones*. HPLC-MS analysis was carried out by Secondary Military Medical School College of Pharmacy. ACN and water 0.1% formic acid were used as mobile phases with elution gradient from 5% to 95%. MS/MS was used for component identification.

#### 3.2.9. Safety Issue Assessment

The experiment protocol followed Technical Guiding Principle of Acute Toxicity Research on Traditional Chinese Medicine or Natural Medicine [7, 9, 10]. Briefly, mice were divided into three groups with 3 males and 3 females per group. After fasting for one day, the first group was given 0.8 ml extract at the concentration of 1,000 mg/ml, 200 mg/ml, and 0 mg/ml. All mice were observed for 14 days in terms of daily weight changing, diet, appearance, action, secretion, excretion, death, and toxic reaction. Very weak mice were slaughtered for histopathology analysis before death. On 14th day the rest mice were slaughtered for histopathology analysis.

## 4. Results

### 4.1. The Antibacterial Effect of Nandina Leaf Extract

The antibacterial effect of nandina leaf extract against* Staphylococcus aureus* ATCC25923,* Escherichia coli* ATCC25922,* Streptococcus pyogenes* CMCC(B)32175,* Pseudomonas aeruginosa* ATCC25793, and* Acinetobacter baumannii* JMD80 strains showed that it inhibited the growth of these test bacteria (Table 1).

### 4.2. The Asthma Prevention Effect of Nandina Leaf Extract

The symptoms of guinea pig model of asthma after treatment by nandina leaf extract were shown in Table 2. Asthma model group showed allergic reaction, but not in negative control group. Nandina leaf extract, as positive control aminophylline, could alleviate asthma symptom for some degree.

### 4.3. The Effect of Nandina Leaf Extract on Bacteria Morphology

**Figure 1:**
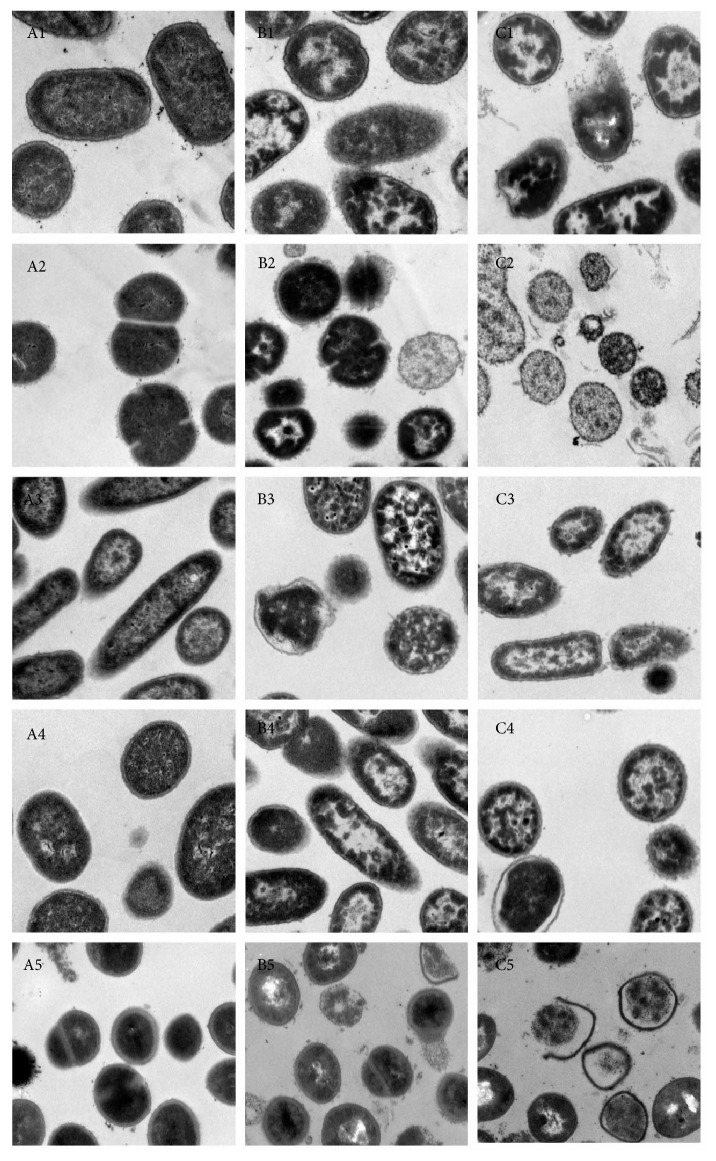
The change of bacterial morphology after nandina leaf extract treatment: A, control; B, 30 min after treatment; C, 60 min after treatment; 1,* E. coli*; 2,* S. pyogenes*; 3,* P. aeruginosa*; 4,* A. baumannii*; 5,* S. aureus*.

*E. coli*,* S. pyogenes*,* P. aeruginosa*,* A. baumannii*, and* S. aureus* were treated by 24 mg/ml nandina leaf extract for 30 min and 60 min and observed under transmission electron microscope. After treatment for 30 min (Figure 1), treated* E. coli* cells were disrupted with pyknosis and incomplete membrane, and the cytoplasm content was spilled, whereas the membrane of untreated* E. coli* was clear with fine structure; after treatment for 60 min, treated* E. coli* became abnormal and almost broke into pieces. The same symptom was found for treated* S. pyogenes*,* P. aeruginosa*,* A. baumannii*, and* S. aureus* cells.

### 4.4. Effective Component Groups of Nandina Leaf Extract

Nandina leaf extract was reextracted by ethanol (extract A), petroleum ether (extract B), and ethyl acetate (extract C). The original leaf extract was named extract D. The antibacterial effects of these groups against* Staphylococcus aureus* ATCC25923, Escherichia coli ATCC25922,* Streptococcus pyogenes* CMCC(B)32175,* Pseudomonas aeruginosa* ATCC25793, and* Acinetobacter baumannii* JMD80 strains were evaluated. The results showed that extract C and extract D can inhibit the growth of these tested bacteria; however, extract A and extract B cannot. As shown in Tables 3 and 4, extract C had the strongest activity on antibacterial and asthma prevention. Therefore, the components group of extract C was analyzed further.

### 4.5. Primary Effective Components Analysis of Extract C

Both Dragendorff's reagent and magnesium hydrochloride tests showing positive effect indicated that both alkaloids and flavones were major component of extract C, with concentration of 68.6% and 56.2%, respectively. Further analysis of these components was carried out.

### 4.6. HPLC-MS/MS Analysis of Effective Components

#### 4.6.1. Alkaloids Analysis in Nandina Extract C

**Figure 2:**
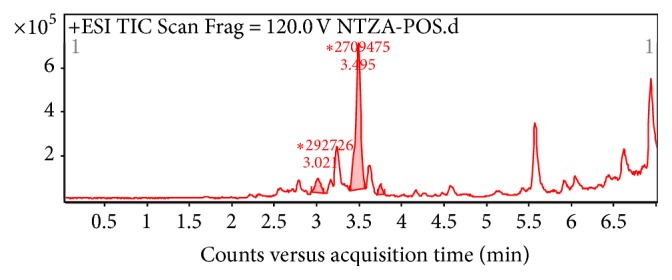
Total ion chromatogram of alkaloids in nandina leaf extract.

**Figure 3:**
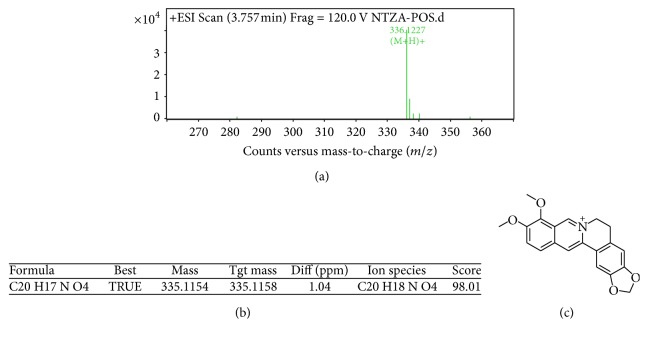
MS/MS chromatogram of berberine in nandina leaf extract (a); molecular ion peak (b); molecular structure (c).

**Figure 4:**
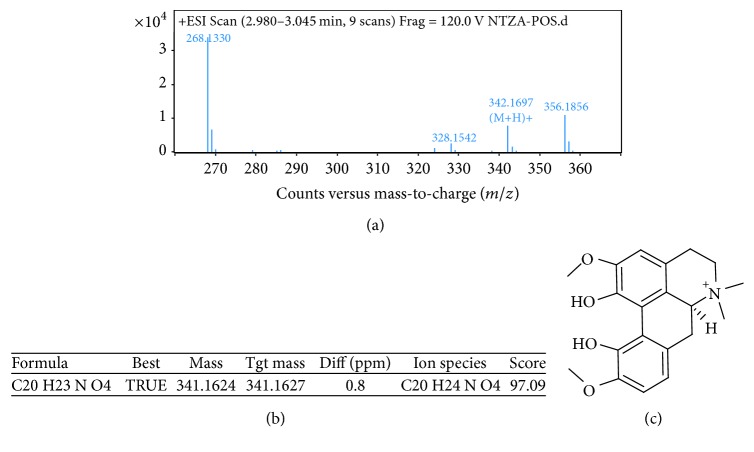
MS/MS chromatogram of magnoflorine in nandina leaf extract (a); molecular ion peak (b); molecular structure (c).

**Figure 5:**
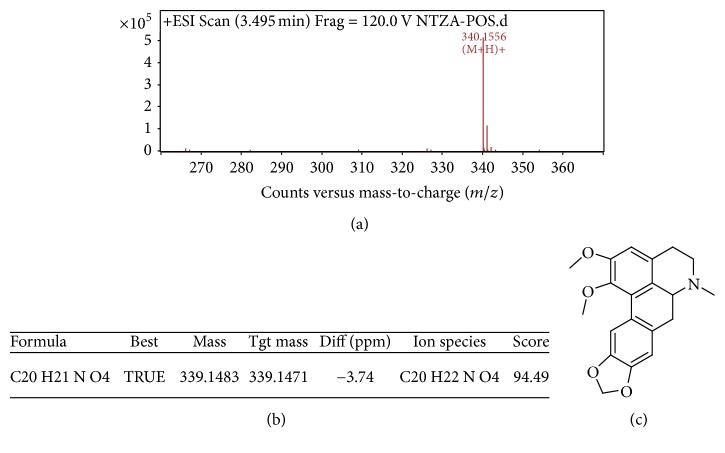
MS/MS chromatogram of O-methyldomesticine in nandina leaf extract (a); molecular ion peak (b); molecular structure (c).

As shown in Figure 2, the three major alkaloids in nandina extract C were berberine (*M* = 336) at 3.757 min, magnoflorine (*M* = 342) at 3.021 min, and O-methyldomesticine (*M* = 339) at 3.495 min; the MS/MS graphs were shown in Figures 3, 4, and 5, respectively.

#### 4.6.2. Flavone Analysis in Nandina Extract C

**Figure 6:**
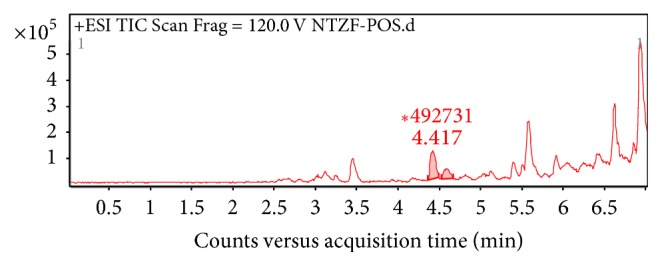
Total ion chromatogram of flavones in nandina leaf extract.

**Figure 7:**
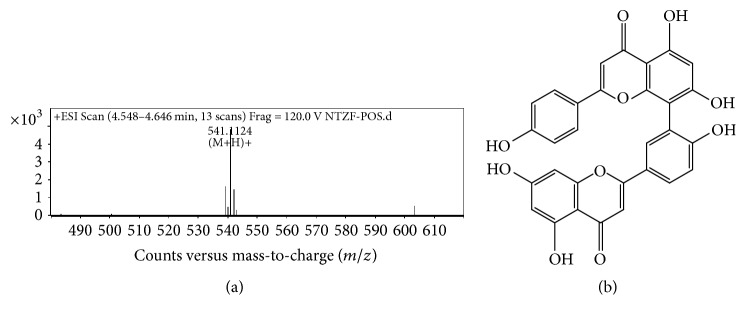
MS/MS chromatogram of amentoflavone in nandina leaf extract (a); molecular structure (b).

**Figure 8:**
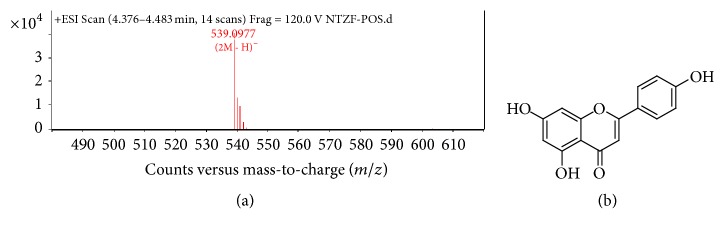
MS/MS chromatogram of apiolin in nandina leaf extract (a); molecular structure (b).

As shown in Figure 6, the two major flavones in nandina extract C were amentoflavone (C30H18O10, *M* = 538) at 4.417 min, Figure 7; apiolin (C15H10O5, *M* = 270) at 4.589 min. The MS/MS graphs were shown in Figures 7 and 8, respectively.

### 4.7. The Antibacterial and Asthma Prevention Effect of Alkaloids and Flavones in Nandina

The antibacterial activities of alkaloids and flavones against* S. aureus ATCC25923, E. coli ATCC25922, S. pyogenes CMCC(B)32175, P. aeruginosa ATCC25793*, and* A. baumannii JMD80* strains were evaluated. As shown in Tables 5 and 6, alkaloids had good effect against Gram-positive bacteria and had good effect on asthma prevention; flavones had good effect against Gram-negative bacteria and had no effect on asthma prevention. This result suggested that alkaloids and flavones were two integrative components against chronic bronchitis.

### 4.8. Safety Issue Evaluation of Nandina Leaf Extract

**Figure 9:**
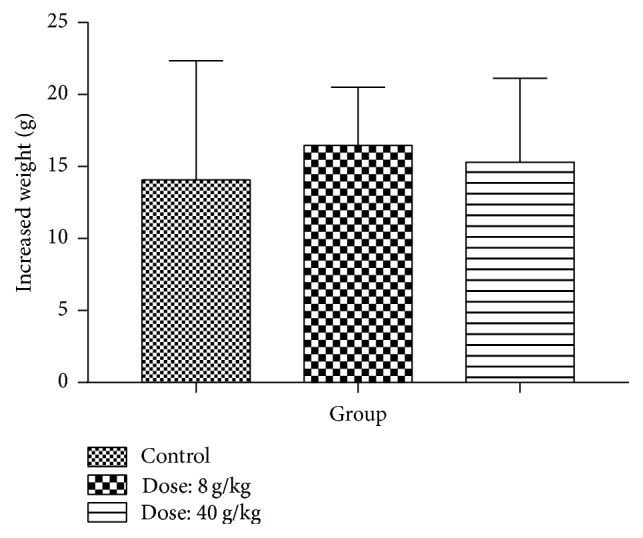
The change of mice weight after 14 days of treatment by nandina leaf extract.

**Figure 10:**
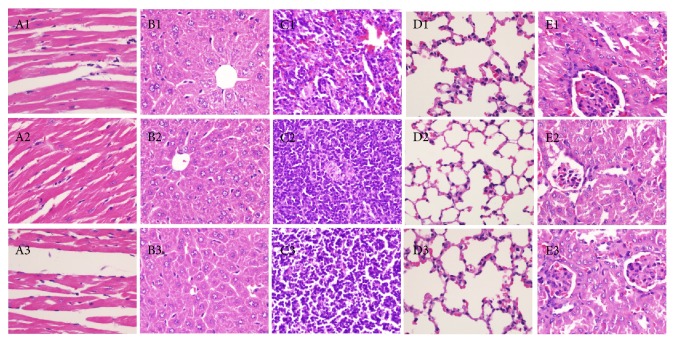
The pathological change of mice tissue (HE, ×400): 1. negative control; 2. 8 g/Kg treatment by nandina leaf extract; 3. 40 g/Kg treatment by nandina leaf extract; A. no change in heart muscle fibers; B. no change in liver cells; C. no change in lymph cells; D. no change in lung tissues; E. no change in glomerulus.

The increasing weight of mice in each group was shown in Figure 9. No obvious weight reduction was found between control group and treated group after 14 days of observation. Further, there were no other difference such as death rate, behavior, and other toxic reaction. Pathology studies on collected heart, spleen, liver, lung, and kidney showed that no changes were found in these tissues (Figure 10).

## 5. Discussion

*Nandina domestica* (heavenly bamboo or sacred bamboo in English), a suckering shrub in the Berberidaceae family, grows in the Shaanxi, Jiangxu, Zhejiang, Anhui, Jiangxi, Fujian, Hubei, Guizhou, and Sichuan Province [2], is harvested in Autumn in China, and is used as a traditional Chinese drug against chronic bronchitis. The key effects against chronic bronchitis are antibacterial and asthma prevention. In this study, we try to find out the antibacterial and asthma prevention effect of nandina leaves and to assess the safety issue.

There are three commonly used methods to test the antibacterial effect of Chinese medicine: (1) solid-phase agar dilution method; (2) liquid medium dilution method; (3) agar diffusion method, including filter paper disk method, punched hole method, and *E*-test. Tang and Liu [11] from Shangdong Agriculture University concludes that solid-phase agar dilution method is the most convenient way to find out minimum MIC, easy to find out contamination and mutation, and has the best repetition. Therefore, solid-phase agar dilution method was used for antibacterial test in this experiment. The result showed that all the five bacteria strains were inhibited by nandina leaf extract to various extents.* S. aureus* ATCC25923 and* P. aeruginosa* ATCC25793 were inhibited most strongly.* P. aeruginosa* is known to be resistant against many antibiotics due to the low permeability of the pump system of the membrane and is the cause of most in-hospital infection. In some departments,* P. aeruginosa* causes the most serious problem and around 10–35% infection is due to* P. aeruginosa* infection [12, 13]. The anti-*P. aeruginosa* effect of nandina leaf extract needs to be further studied. The bacteria morphology after nandina leaf extract treatment was found to be destroyed: (1) cell wall and cell membrane were broken; (2) cytoplasm is constrained and spilled out; (3) the destroying extent was positively related to the time of treatment. In conclusion, nandina leaf extract showed high antibacterial activity through destroying the cell wall and changing the cell membrane permeability. The underlying mechanism needs to be studied further.

Asthma is a kind of chronic bronchitis inflammation, causing repetitive gasp, short breath, chest choke, and/or cough and happens mostly in the evening and/or in the early morning when trachea increases the sensitivity to many stimulators. Many cells are involved, including mastocyte, acidic granulocyte macrophage, and T lymphocyte. Symptom alleviates by itself or after treatment. In the past decade, the affection rate and the death rate of asthma increase in the US, Great Britain, Australia, New Zealand, and so forth. There are about 100 million asthma patients worldwide and asthma has become a serious problem to public health. In China, more than 10 million people (1%) are affected by asthma and for children 3%. Our result showed that nandina leaf extract has the same effect as aminophylline in asthma prevention effect.

In order to find out which are the active components in nandina leaf extract, we reextract the nandina leaf extract with solvents of different polarity, ethanol, petroleum ether, and ethyl acetate, to test their ability to inhibit the growth of bacteria and asthma prevention effect. Compared with the water soluble phase, the ethyl acetate phase can effectively inhibit the growth of bacteria, whereas the extracts of the other two phases cannot inhibit the growth of bacteria. This result showed that the effective components of nandina leaves resided in ethyl acetate phase. We further found that the active component groups in ethyl acetate phase were alkaloids and flavones. Alkaloids are a group of natural chemical compounds produced by a large variety of organisms, including bacteria, fungi, plants, and animals (also called secondary metabolites). They often have pharmacological effects and are used as medications or as recreational drugs or in entheogenic rituals and so on. Further study on alkaloid contained in the nandina leaf extract can further explain why it can treat the asthma and inhibit the pathogen. Another important result of our research is that it can provide a novel treatment of superbacteria* S. aureus*, especially clinical multidrug-resistant organisms.

HPLC-MS/MS analysis further confirmed that alkaloids in ethyl acetate phase contained O-methyldomesticine, which is a known to be effective in releasing the asthma. Therefore, we can suppose that the reason why nandina extract can release asthma is that it contains O-methyldomesticine.

Most of Berberidaceae are toxic to human, so it is necessary to evaluate the safety issue of nandina to human and animal. The result suggested that the maximum dose of nandina at 40 g/kg showed no obvious toxic effect on mice. 14 days of continuous observation was carried out on mice weight, food intake, appearance, secretion, and feces. Mice were slaughtered on 14th day and heart, lung, kidney, and lung were analyzed. No toxic effect was found. However, MTD and LD50 would be found out in further studies.

## Figures and Tables

**Table 1:** Antibacterial effect of nandina leaf extract.

Bacteria	MIC (mg/ml)
Gram-positive	
*S. pyogenes*	3
*S. aureus*	6
Gram-negative	
*E. coli*	12
*P. aeruginosa*	6
*A. baumannii*	12

**Table 2:** Asthma prevention of nandina leaf extract.

Symptom class	Negative control	Asthma model	Positive control (2 mg/kg)	Nandina leaf extract (1 g/kg)
I (no response)	6	0	5	4
II (dyspnea)	0	2	1	2
III (cough)	0	3	0	0
IV (death)	0	1	0	0

**Table 3:** The antibacterial effect of nandina leaf extract against 5 bacteria (MIC).

Bacteria	MIC (mg/ml)			
	Total leaf extract	Petroleum ether	Ethyl acetate	Nandina ethanol
Gram-positive				
*S. pyogenes*	3	≥24	1.5	≥24
*S. aureus*	6	≥24	6	≥24
Gram-negative				
*E. coli*	12	≥24	6	≥24
*P. aeruginosa*	6	≥24	3	≥24
*A. baumannii*	12	≥24	3	≥24

**Table 4:** The asthma prevention effect of nandina leaf extract.

Symptom class	Negative control	Guinea pig model of asthma	Positive control	Petroleum ether (mg/kg)		Ethyl acetate (mg/kg)		Nandina ethanol (mg/kg)	
				50	25	50	25	50	25
I	6	0	5	1	1	5	4	0	0
II	0	2	1	3	1	1	2	3	1
III	0	3	0	2	4	0	0	3	4
IV	0	1	0	0	0	0	0	0	1

**Table 5:** The antibacterial effect of alkaloids and flavones in nandina leaf extract (MIC).

Bacteria	MIC (mg/ml)	
	Alkaloids	Flavones
Gram-positive		
*S. pyogenes*	1.25	5
*S. aureus*	0.156	5
Gram-negative		
*P. aeruginosa*	2.5	1.25
*A. baumannii*	2.5	1.25
*E. coli*	10	2.5

**Table 6:** The asthma prevention effect of alkaloids and flavones in nandina leaf extract.

Symptom class	Negative control	Guinea pig model of asthma	Positive control	Alkaloids (mg/kg)		Flavones (mg/kg)	
				10	5	10	5
I	6	0	5	5	4	1	1
II	0	2	1	1	2	3	1
III	0	3	0	0	0	2	4
IV	0	1	0	0	0	0	0

## References

[1] Chen M. Pathogenic analysis of chronic bronchitis sputum by culture. *Chinese Journal of Practical Medicine*.

[2] Liu J. The utilization and development research of nandina resource. *Chinese Wild Plant Resources*.

[3] Bajpai V. K., Yoon J. I., Kang S. C. (2009). Antifungal potential of essential oil and various organic extracts of *Nandina domestica* Thunb. against skin infectious fungal pathogens. *Applied Microbiology and Biotechnology*.

[4] Wilson R., Grossman R. (2000). Introduction: The role of bacterial infection in chronic bronchitis. *Seminars in Respiratory Infections*.

[5] Wilson R. (2000). Evidence of bacterial infection in acute exacerbations of chronic bronchitis. *Seminars in Respiratory Infections*.

[6] Dai K. Prescription of cough treatment. *Shanxi Traditional Chinese Medicine*.

[7] Bajpai V. K., Rahman A., Kang S. C. (2008). Chemical composition and inhibitory parameters of essential oil and extracts of Nandina domestica Thunb. to control food-borne pathogenic and spoilage bacteria. *International Journal of Food Microbiology*.

[8] Liu C. Treatment of whooping cough by Bamboo pocket bell Soup. *Zhejiang Traditional Chinese Medicine*.

[9] Gui H., Tang J., Shuying C., Hu S. Clinical Observation of Fufang nandina injection on gastroenteritis of pig. *Chinese Journal of Veterinary Medicine*.

[10] Tsukiyama M., Akaishi T., Ueki T., Okumura H., Abe K. (2007). The extract from Nandina domestica Thunberg inhibits histamine- and serotonin-induced contraction in isolated guinea pig trachea. *Biological & Pharmaceutical Bulletin*.

[11] Tang L., Liu C. Comparison of detection methods of Antibacterial of TCM. *Chinese Medicine Science and Technology*.

[12] Matsumoto T., Fujita M., Hirano R. (2016). Chronic Pseudomonas aeruginosa infection-induced chronic bronchitis and emphysematous changes in CCSP-deficient mice. *International Journal of Chronic Obstructive Pulmonary Disease*.

[13] Khan J. A., Iqbal Z., Rahman S. U. Report: prevalence and resistance pattern of Pseudomonas aeruginosa against various antibiotics. *Pakistan Journal of Pharmaceutical Sciences*.

